# Immunotherapy Improves the Survival of Stage 4 Non–Small Cell Lung Cancer Patients at the US Population Level: The Real‐World Evidence

**DOI:** 10.1111/crj.70000

**Published:** 2024-09-14

**Authors:** Yuxuan Wei, Rui Zhang, Ruikang Yin, Shijie Wang, Jianglong Han, Ruyan Chen, Zhenming Fu

**Affiliations:** ^1^ Cancer Center Renmin Hospital of Wuhan University Wuhan China; ^2^ College of Basic Medicine Zhengzhou University Zhengzhou China; ^3^ Radiation Oncology Department, China‐Japan Friendship Hospital Chinese Academy of Medical Sciences & Peking Union Medical College Beijing China

**Keywords:** age‐period‐cohort analysis, immunotherapy, Joinpoint survival model, lung cancer, tyrosine kinase inhibitors

## Abstract

**Introduction:**

Immunotherapy has revolutionized the management of lung cancer and improved lung cancer survival in trials, but its real‐world impact at the population level remains unclear.

**Methods:**

Using data obtained from eight Surveillance, Epidemiology, and End Results (SEER) registries from 2004 through 2019, we addressed the long‐term trends in the incidence, incidence‐based mortality (IBM), and survival of lung cancer patients in the United States.

**Results:**

The incidence and IBM of both non–small cell lung cancer (NSCLC) and small cell lung cancer (SCLC) all significantly decreased steadily from 2004 to 2019. The 1‐year survival (1‐YS) of both NSCLC and SCLC improved over time, with the best improvement observed for Stage 4 NSCLC. Two significant turning points of Stage 4 NSCLC 1‐YS were observed over the years: 0.63% (95% confidence interval [CI]: 0.33%–0.93%) from 2004 to 2010, 0.81% (95% CI: 0.41%–1.21%) from 2010 to 2014 and a striking 2.09% (95% CI: 1.70%–2.47%) from 2014 to 2019. The same two turning points in 1‐YS were pronounced for Stage 4 NSCLC in women, which were coincident with the introduction of epidermal growth factor receptor tyrosine kinase inhibitors (EGFR‐TKIs) and immunotherapy. However, for Stage 4 NSCLC in men, only one significant turning point in the 1‐YS starting in 2014 was found, which might only correspond to immunotherapy. Significant period effects in reduced IBM were also observed for both Stage 4 AD and Stage 4 SQCC during the period.

**Conclusion:**

This SEER analysis found that immunotherapy improved the survival of Stage 4 NSCLC patients at the population level in the United States. This real‐world evidence confirms that immunotherapy has truly revolutionized the management of lung cancer.

## Graphical abstract

This study explored whether immunotherapy improved prognosis for lung cancer patients at the population level from 2004 to 2019. The introduction of tyrosine kinase inhibitors and immunotherapy resulted in two significant increases in the 1‐year survival of Stage 4 NSCLC during the past 15 years. The real‐world evidence confirms that immunotherapy has revolutionized the management of lung cancer.

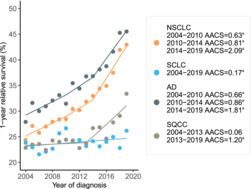

## 1 Introduction

Although lung cancer incidence has declined in the United States in recent years [1], a disease including non–small cell lung cancer (NSCLC) and small cell lung cancer (SCLC), remains the number 1 cause of cancer‐related death [2]. Over the past 20 years, significant improvements have been made in NSCLC treatment with the advent of targeted therapies [3, 4, 5, 6] for genetic alterations, including but not limited to epidermal growth factor receptor (EGFR) mutations and anaplastic lymphoma kinase (ALK) rearrangements [7]. For advanced lung cancer without genetic driver mutations, immunotherapy has soon emerged as an efficacious treatment option. Currently, an immune checkpoint inhibitor (ICI)–based regimen alone or in combination with chemotherapy is the preferred first‐line option for Stage 4 NSCLC, and extensive‐stage SCLC since nivolumab was approved by the Food and Drug Administration (FDA) in 2015 for patients with squamous NSCLC [8] and atezolizumab in 2018 for SCLC [9].

Recently, Howlader et al. suggested that a reduction in incidence along with treatment advances—particularly approvals for and use of targeted therapies—is likely to explain the reduction in mortality observed during 2013–2016 [10]. These advancements may have contributed to population‐level improvement in NSCLC overall survival (OS). NSCLC has long been classified as adenocarcinoma (AD), squamous cell carcinoma (SQCC), and large cell carcinoma (LCC), as well as other specified carcinomas and unspecified types [11]. Although dynamic changes in lung cancer incidence and mortality are well documented, little is known regarding survival trends by histological type [11].

Immunotherapy has recently been shown to bring clinically meaningful long‐term OS benefits for late‐stage NSCLC in large Phase III clinical trials [12, 13]. These trends, if they can be confirmed in real‐life at the population level, would have important implications for clinical care and national policy around NSCLC. Therefore, the present study sought to examine how the incidence, incidence‐based mortality (IBM), and survival changes of lung cancer by all histological types over the past two decades, with a focus on evaluating the possible impact of immunotherapy on patient survival at the population level.

## 2 Methods

### 2.1 Data Source

**FIGURE 1:**
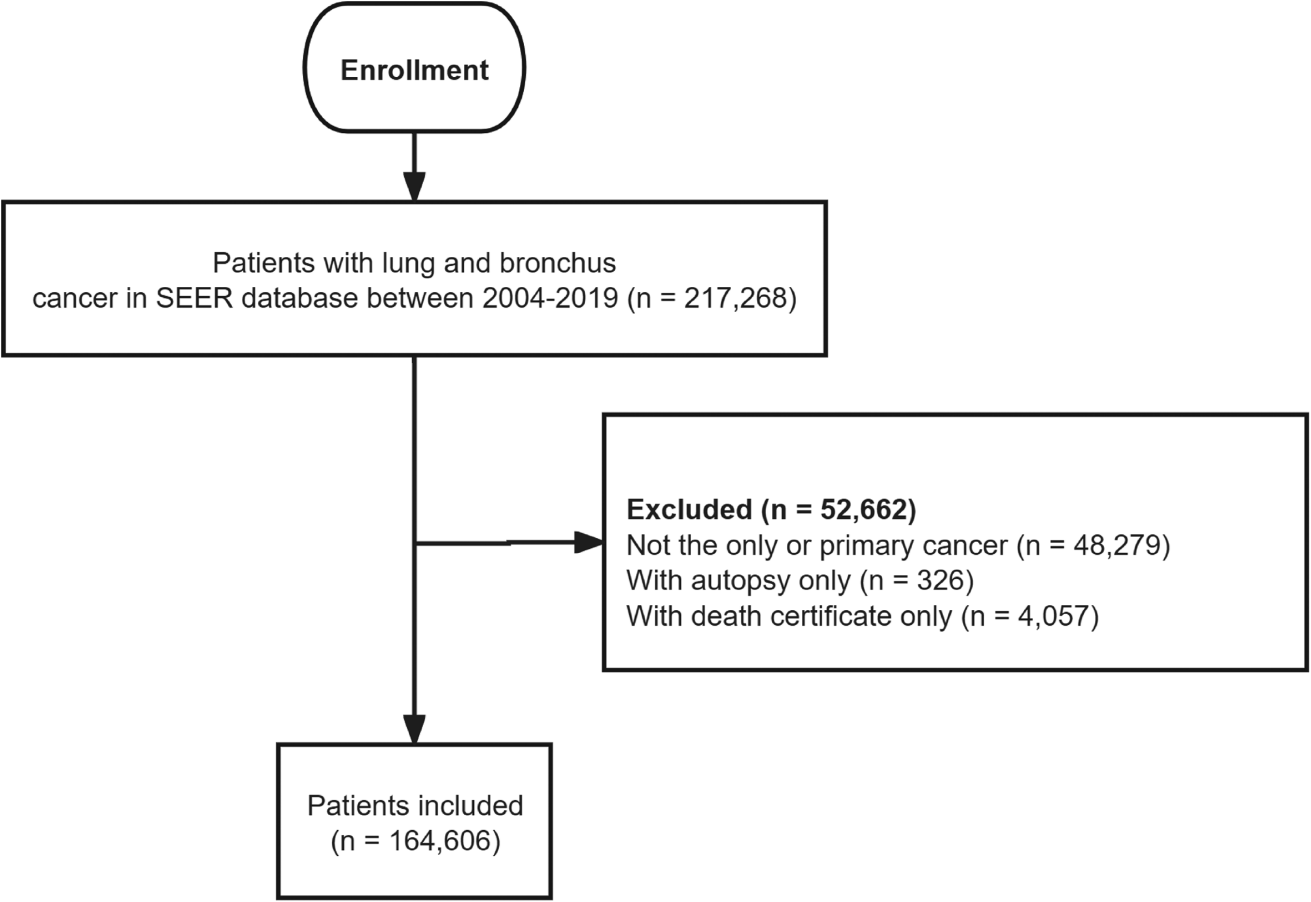
The flowchart of study population selection. Abbreviation: SEER, Surveillance, Epidemiology, and End Results.

The National Cancer Institute (NCI) Surveillance, Epidemiology, and End Results (SEER) program is a unique real‐life source for historical population‐based cancer data of the United States [14], which covers 47.9% of the US population [15]. Patients with lung and bronchial cancer were selected from the SEER‐8 database using SEER*Stat software (Version 8.4.2; NCI). The main histologic type categories were defined according to the classification system of Lewis et al. [11], in which histological groups were created based on the International Classification of Diseases for Oncology 3rd Edition (ICD‐O‐3) morphology codes. The morphology codes were as follows: NSCLC (8003‐4, 8012‐5, 8021‐2, 8030‐5, 8046, 8050‐2, 8070‐6, 8078, 8082‐4, 8090, 8094, 8120, 8123, 8140‐1, 8143‐5, 8147, 8190, 8200‐1, 8211, 8240‐1, 8243‐6, 8249, 8250‐5, 8260, 8290, 8310, 8320, 8323, 8333, 8401, 8430, 8440, 8470‐1, 8480‐1, 8490, 8503, 8507, 8525, 8550, 8560, 8562, 8570‐2, 8574‐6); SCLC (8002, 8041‐5); AD (8015, 8050, 8140‐1, 8143‐5, 8147, 8190, 8201, 8211, 8250‐5, 8260, 8290, 8310, 8320, 8323, 8333, 8401, 8440, 8470‐1, 8480‐1, 8490, 8503, 8507, 8550, 8570‐2, 8574, 8576); SQCC (8051‐2, 8070‐6, 8078, 8083‐4, 8090, 8094, 8120, 8123). We used 2004 as the first year in the analysis period because the World Health Organization (WHO) revised the histological classification of lung cancer in 2004, which covered the molecular biology of the various lung malignancies and provided standardized nomenclature and diagnostic criteria for lung cancer histology [16]. Patients who were only diagnosed by death certificates or autopsy were excluded for incomplete histology subtype information. Patients whose lung cancer was not the only or primary cancer were also excluded (resulting in the exclusion of 24.2% of patients overall) (Figure 1). The lung cancer incidence from 2004 through 2019 was calculated after accounting for reporting delay [17]. As IBM includes deaths among incident cases diagnosed in previous years, cases diagnosed a number of years ago need to be followed up. Considering the aggressiveness of lung cancer, a 5‐year follow‐up period would be sufficient to calculate reliable IBM [18].

### 2.2 Statistical Methods

We report the incidence and IBM according to calendar year, sex, and subtype. The incidence and IBM rates were age‐adjusted to the 2000 US standard population and expressed per 100 000 person‐years. We used Joinpoint software (Version 4.9.1.0), a statistical software program developed specifically for cancer surveillance by NCI, to characterize piecewise log‐linear time calendar trends in the age‐standardized rates by sex and cancer subtype (https://surveillance.cancer.gov/joinpoint/download) [19, 20]. To characterize trends in incidence and IBM rates over time, we applied the best‐fitting log‐linear regression model to calculate the annual percent change (APC) and corresponding 95% confidence intervals (CIs) of each line segment and identified the joinpoints when APCs changed significantly (*p* < 0.05) [20]. Two‐tailed *t*‐tests were used to assess whether APCs were significant [20]. Meanwhile, we provide trends of 1‐year relative survival (1‐YS) among patients with lung cancer according to sex, subtype, and calendar year using JPSurv software, which is developed by NCI to analyze trends in survival with respect to year of diagnosis (https://analysistools.cancer.gov/jpsurv/) [21]. The annual absolute change in survival (AACS) and corresponding 95% CIs were calculated through a joinpoint survival model to characterize 1‐YS trends. CIs were calculated using the delta method [21].

Age‐period‐cohort model is a fundamental model that is used to analyze population‐based registry data and describes the mathematical associations among the rate of cancer and age, calendar period, and birth cohort. We assessed the relationships by using NCI's age‐period‐cohort analysis web tool (https://analysistools.cancer.gov/apc/) [22]. The IBM of Stage 4 lung cancer was calculated using eight 5‐year age groups (45–49, 50–54, and 80–84) and three corresponding calendar periods (2005–2009, 2010–2014, and 2015–2019). Because some data on Stage 4 lung cancer patients younger than 45 years of age were missing from the SEER database, people older than 45 years of age were used for the analysis. The rate ratio (RR) of IBM in any given calendar period (or birth cohort) versus a referent period (or birth cohort) was calculated and adjusted for age and nonlinear cohort (or period) effects. The age‐period‐cohort model used Wald chi‐square tests to test the significance of the RR [22].

## 3 Results

Between 2004 and 2019, we identified a total of 164 606 patients diagnosed with lung and bronchial cancer in the SEER‐8 database. Table 1 presents the baseline characteristics of the patients we selected, including demographics, clinical characteristics, and treatment. In terms of the entire study cohort, there were 85 252 males and 79 354 females. The median age was 70 years. White (80.0%) and NSCLC (86.6%) accounted for predominance. With regard to the stage of lung cancer, the most was the Stage 4 disease, comprising approximately 46.4% of patients, followed by the Stage 3 (21.4%), Stage 1 (18.4%) and Stage 2 (4.8%). For Stage 4 patients, in addition to chemotherapy, which was the most common therapy (43.5%), more and more patients had also received radiotherapy (38.6%).

**TABLE 1:** Baseline characteristics of lung and bronchus cancer patients in the SEER‐8 database, 2004–2019.

Characteristic	Cases (%)
Total	164 606
Age (median)	70
Race	
White	131 746 (80.0)
Black	14 185 (8.6)
Other (American Indian/AK Native, Asian/Pacific Islander)	18 322 (11.1)
Unknown	353 (0.2)
Sex	
Female	79 354 (48.2)
Male	85 252 (51.8)
Histology	
NSCLC	142 619 (86.6)
SCLC	20 480 (12.4)
Unknown	1507 (0.9)
Grade	
1	8038 (4.9)
2	22 915 (13.9)
3	33 208 (20.2)
4	4850 (2.9)
Unknown	95 595 (58.1)
Stage	
Stage 1	30 358 (18.4)
Stage 2	7830 (4.8)
Stage 3	35 245 (21.4)
Stage 4	76 360 (46.4)
Unknown	14 813 (9.0)
Surgery	
Yes	33 601 (20.4)
No	130 089 (79.0)
Unknown	916 (0.6)
Chemotherapy	
Yes	71 626 (43.5)
No/unknown	92 980 (56.5)
Radiotherapy	
Yes	63 521 (38.6)
None/unknown	10 1085 (61.4)

Abbreviations: NSCLC, non‐small cell lung cancer; SCLC, small cell lung cancer.

**FIGURE 2:**
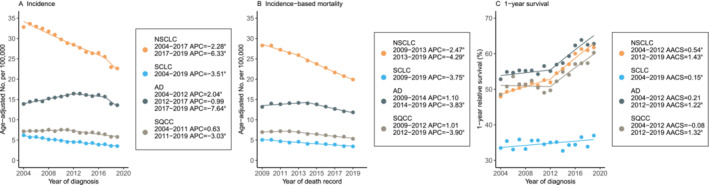
Lung cancer incidence, incidence‐based mortality, and survival trends by histological types. Abbreviations: AACS, average absolute change in survival; AD, adenocarcinoma; APC, annual percentage change; NSCLC, non‐small cell lung cancer; SCLC, small cell lung cancer; SQCC, squamous cell carcinoma. Line segments of each curve were selected with the Joinpoint regression program (Panels A and B) and JPsurv analysis tool (Panel C). ^a^ *p* < 0.05.

The trends in lung cancer incidence, IBM, and survival are presented in Figure 2. Overall, the lung cancer incidence and IBM decreased steadily from 2004 to 2019 and from 2009 to 2019. For NSCLC, the incidence decreased gradually, by −2.28% annually (95% CI, −2.56% to −1.99%) from 2004 through 2017 and then more steeply, by −6.33% annually (95% CI, −11.47% to −0.88%) from 2017 through 2019. IBM decreased by −2.47% annually (95% CI, −3.34% to −1.59%) from 2009 through 2013 and then decreased more quickly, by −4.29% annually (95% CI, −4.76% to −3.82%) from 2013 through 2019. Similar declining trends of incidence and IBM were observed for NSCLC subtypes AD and SQCC. From 2004 to 2019, one significant turning point in 2012 was found for the 1‐YS rate of NSCLC patients. As a result, the improved 1‐YS was categorized into two significant segments: It was improved by 0.54% (95% CI, 0.37%–0.71%) per year from 2004 to 2012 and then showed a much faster annual increase of 1.43% (95% CI, 1.24%–1.61%) from 2012 to 2019. Similar trends of survival improvement over time were found for the AD and SQCC subtypes. On the other hand, the incidence and IBM trends of SCLC decreased steadily by −3.51% (95% CI, −3.82% to −3.20%) and − 3.75% (95% CI, −4.54% to −2.95%), respectively, whereas the 1‐YS rate improved smoothly but significantly by 0.15% (95% CI, 0.01%–0.29%) over the study period.

**FIGURE 3:**
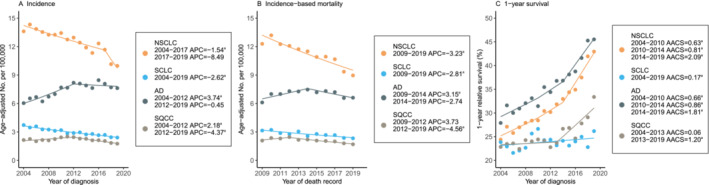
Stage 4 lung cancer incidence, incidence‐based mortality, and survival trends by histological types. Abbreviations: NSCLC, non–small cell lung cancer; SCLC, small cell lung cancer; AD, adenocarcinoma; SQCC, squamous cell carcinoma; APC, annual percentage change; AACS, average absolute change in survival. Line segments of each curve were selected with the Joinpoint regression program (Panels A and B) and JPsurv analysis tool (Panel C). ^a^ *p* < 0.05.

Similarly, the incidence and IBM for both Stage 4 NSCLC (including AD and SQCC) and Stage 4 SCLC declined over the study period (Figure 3). The incidence of Stage 4 NSCLC decreased by −1.54% annually (95% CI, −2.04% to −1.05%) from 2004 through 2017 and then by −8.49% annually (95% CI, −17.03% to 0.94%) from 2017 through 2019. IBM decreased by −3.23% annually (95% CI, −4.15% to −2.31%) from 2009 through 2019. Stage 4 SCLC incidence and IBM trends decreased steadily by −2.62% (95% CI, −2.96% to −2.27%) and −2.81% (95% CI, −3.87% to −1.74%), respectively, whereas the 1‐YS rate increased by 0.17% (95% CI, 0.04%–0.30%). However, two significant turning points were observed in the annual increases in 1‐YS for Stage 4 NSCLC over the years. Namely, three significant increases in 1‐YS were observed over the years: 0.63% (95% CI, 0.33%–0.93%) from 2004 to 2010, 0.81% (95% CI, 0.41%–1.21%) from 2010 to 2014, and 2.09% (95% CI, 1.70%–2.47%) from 2014 to 2019. The 1‐YS rate of AD demonstrated a similar trend, whereas the 1‐YS of Stage 4 SQCC increased by 1.20% (95% CI, 0.8%–1.59%) from 2013 to 2019 after a plateau from 2004 to 2013.

**FIGURE 4:**
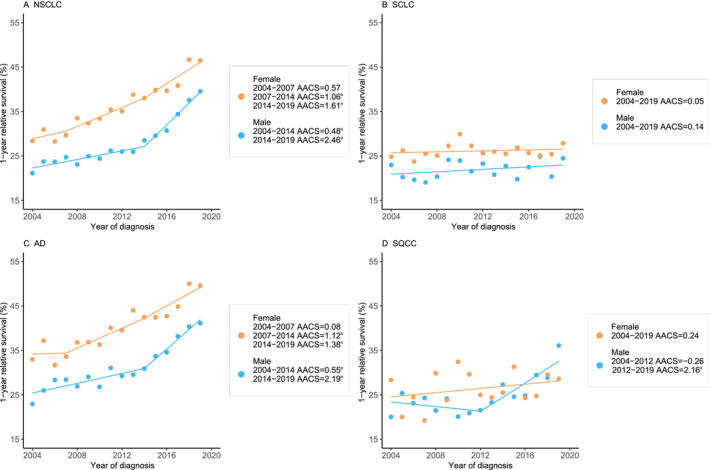
1‐year survival trends of Stage 4 lung cancer among female and male by histological types. Abbreviations: AACS, average absolute change in survival; AD, adenocarcinoma; NSCLC, non–small cell lung cancer; SCLC, small cell lung cancer; SQCC, squamous cell carcinoma. Line segments of each curve were selected with the JPsurv analysis tool. ^a^ *p* < 0.05.

Figure 4 and Table 2 further illustrate the 1‐YS rate of Stage 4 lung cancers by sex. For Stage 4 NSCLC, 1‐YS increased by 0.48% (95% CI, 0.33%–0.63%) per year in 2004–2014 and by 2.46% (95% CI, 2.10%–2.81%) per year in 2014–2019 among men. Two significant turning points of 1‐YS were observed among women: 0.57% (95% CI, −0.53% to 1.68%) annually in 2004–2007, 1.06% (95% CI, 0.68%–1.44%) annually in 2007–2014, and a striking 1.61% (95% CI, 1.03%–2.20%) per year in 2014–2019. The 1‐YS trends for Stage 4 AD were similar to those of Stage 4 NSCLC. The 1‐YS increased per year by 0.55% (95% CI, 0.33%–0.78%) in 2004–2014 and by 2.19% (95% CI, 1.73%–2.65%) in 2014–2019 among men. The 1‐YS improved annually by 1.12% (95% CI, 0.61%–1.62%) from 2007 to 2014 and by 1.38% (95% CI, 0.69%–2.08%) from 2014 to 2019 after a plateau from 2004 to 2007 among women. For Stage 4 SQCC, the 1‐YS plateaued among men and women in 2004–2012 and 2004–2019, respectively, and then increased substantially by 2.16% (95% CI, 1.06%–2.16%) yearly from 2012 to 2019 among men. For SCLC in Stage 4, the 1‐YS remained stable over the years among both sexes.

**TABLE 2:** 1‐year survival trends of Stage 4 lung cancer by histological types.

Characteristic		Trends for 1‐year survival (n = 76 360)								
		Trend 1			Trend 2			Trend 3		
		Years	AACS (95% CI)	*p*	Years	AACS (95% CI)	*p*	Years	AACS (95% CI)	*p*
NSCLC	Male	2004–2014	0.48 (0.33–0.63)	< 0.05	2014–2019	2.46 (2.10–2.81)	< 0.05	—	—	—
	Female	2004–2007	0.57 (−0.53–1.68)	0.32	2007‐2014	1.06 (0.68–1.44)	< 0.05	2014‐2019	1.61 (1.03–2.20)	< 0.05
SCLC	Male	2004–2019	0.14 (−0.07–0.34)	0.18	—	—	—	—	—	—
	Female	2004–2019	0.05 (−0.11–0.22)	0.56	—	—	—	—	—	—
AD	Male	2004–2014	0.55 (0.33–0.78)	< 0.05	2014–2019	2.19 (1.73–2.65)	< 0.05	—	—	—
	Female	2004–2007	0.08 (−1.57–1.72)	0.93	2007–2014	1.12 (0.61–1.62)	< 0.05	2014‐2019	1.38 (0.69–2.08)	< 0.05
SQCC	Male	2004–2012	−0.26 (−0.75–0.23)	0.30	2012–2019	1.61 (1.06–2.16)	< 0.05	—	—	—
	Female	2004–2019	0.24 (−0.18–0.65)	0.26	—	—	—	—	—	—

Abbreviations: AACS, average absolute changes in survival; AD, adenocarcinoma; NSCLC, non‐small cell lung cancer; SCLC, small cell lung cancer; SQCC, squamous cell carcinoma.

**FIGURE 5:**
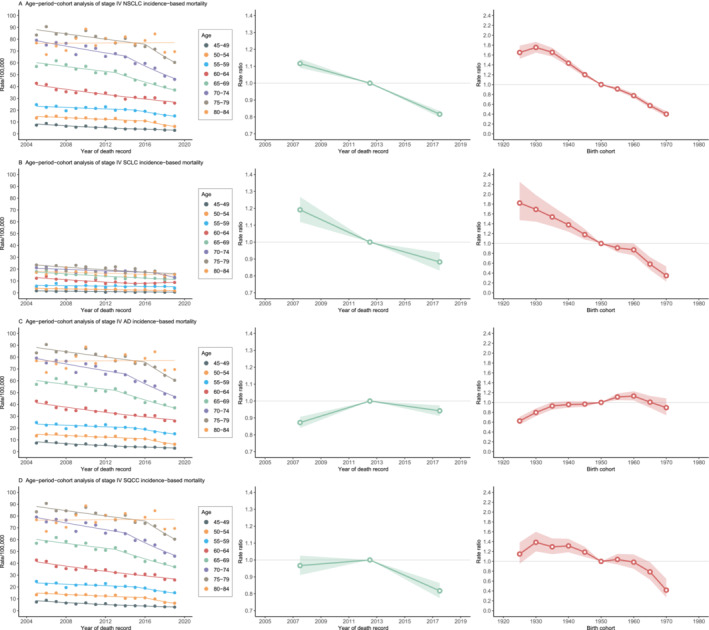
Age‐period‐cohort analyses of Stage 4 lung cancer incidence‐based mortality by histological types. Abbreviations: AD, adenocarcinoma; NSCLC, non–small cell lung cancer; SCLC, small cell lung cancer; SQCC, squamous cell carcinoma. The age‐specific analysis, period rate ratio, and birth cohort rate ratio of Stage 4 lung cancer incidence‐based mortality. Gray zone indicates 95% CIs.

The age‐period‐cohort analysis of the IBM is demonstrated in Figure 5. For different histological subtypes of Stage 4 lung cancer, the IBM of different age groups generally showed a flat or decreasing trend, except for AD, which first increased and then decreased in all age groups older than 50. There was a notable period effect during 2010–2014 regarding the IBM of Stage 4 AD and SQCC. For AD, the period RR in IBM declined during 2015–2019 (RR = 0.94, 95% CI, 0.91–0.97) compared to that of the reference period 2010–2014, after rising trends of RR in IBM over the period 2005–2009. For SQCC, the period RR in IBM during 2005–2009 did not change significantly compared with that during 2010–2014 (RR = 0.97, 95% CI, 0.91–1.03), whereas the RR in IBM of 2015–2019 (RR = 0.82, 95% CI, 0.77–0.86) decreased substantially. The cohort RR in the IBM decreased with increasing birth year in all histological subtypes except Stage 4 AD. The RR of AD first increased and then began to decline around 1960.

## 4 Discussion

In this study, we investigated the temporal changes in lung cancer from 2004 to 2019, focusing on patient survival at the US population level. We observed that the 1‐YS for NSCLC patients increased significantly at two turning points: one in 2010 and another in 2014. These two turning points were more significant for female and AD patients, especially among those with Stage 4 disease. However, for male and SQCC patients, the most remarkable improvement in survival was observed since 2014. These turning points coincided well with the prime time for molecular targeted therapy (since 2009) and the introduction of immunotherapy (2015) and were validated by significant period effects found in the IBM in age‐period‐cohort analyses.

Overall, our findings suggest that the prognosis of lung cancer improved over time at the population level. The turning time points found in NSCLC, especially for Stage 4 disease, coincided with the implementation of gene testing and the introduction of molecular targeted therapy and immunotherapy, respectively. This is consistent with previous studies that reported improved survival outcomes for NSCLC patients treated with platinum‐based chemotherapy, molecular targeted therapy, and immunotherapy [23, 24, 25]. However, the current study is the first to demonstrate the significantly improved survival of NSCLC patients by immunotherapy at the population level.

The substantial improvements in 1‐YS since 2012, especially for women and Stage 4 AD patients, can largely be attributed to the development of drugs that target specific driver gene abnormalities [3, 4, 5, 6]. This notion was further supported by the fact that we did not find the same significant time point for male Stage 4 NSCLC patients, neither for overall nor for AD and SQCC patients. This can be expected because these patients harbored fewer driver gene abnormalities. In 2009, the landmark IPASS study demonstrated that gefitinib was superior to carboplatin/paclitaxel with regard to progression‐free survival in patients with this phenotype, which was driven by EGFR mutation [23]. The median OS was 27.7 months in the gefitinib group and 26.6 months in the carboplatin/paclitaxel group [26]. And gefitinib, as compared with carboplatin/paclitaxel, was associated with a lower rate of Grade 3 or 4 adverse events (28.7% vs. 61.0%), a lower rate of adverse events leading to discontinuation of the drug (6.9% vs. 13.6%), and a lower rate of dose modification due to toxic effects (16.1% vs. 35.2% for carboplatin and 37.5% for paclitaxel) [23]. The IPASS trial has since symbolized the prime time for molecular targeted therapy [27].

In the current study, the best improvement in survival was observed in Asian or Pacific Islander patients with AD, which might be due to more prevalent driver gene mutations in these populations [28]. New generations of EGFR‐TKIs and various TKIs for ALK alterations were subsequently developed for NSCLC patients, with driver gene alterations [29, 30]. The median OS was 26.8 months in osimertinib group and 22.5 months in the platinum/pemetrexed group [31]. Osimertinib was associated with a lower rate of Grade 3 or 4 adverse events (23% vs. 47%) and a lower rate of adverse events leading to discontinuation of the drug (7% vs. 10%) compared with platinum/pemetrexed [31]. The median OS was not reached in the ALK inhibitor crizotinib group and 47.5 months in the chemotherapy (pemetrexed plus cisplatin or carboplatin) group. Survival probabilities 4 years were 56.6% with crizotinib and 49.1% with chemotherapy [32]. The median duration of treatment was 10.9 months in the crizotinib group and 4.1 months in the chemotherapy group, although the rate of adverse events leading to discontinuation of the drug was 5% with crizotinib and 8% with chemotherapy [29]. These facts were consistent with our finding that showed continuous improvement in survival for NSCLC, especially Stage 4 AD, over recent years. The survival trends observed in this study can be mutually corroborated by Howlader's study [10].

In addition, the second significant turning point was found in approximately 2014, which represented even greater and substantial improvements in 1‐YS, especially for women and Stage 4 NSCLC patients. The survival trends of Stage 4 NSCLC, AD, and SQCC all exhibited a turning point in approximately 2014, although this turning point was the second one for AD but the only one for SQCC and male Stage 4 NSCLC patients. In October 2015, the US FDA approved the PD‐1 inhibitor nivolumab, the first immunotherapy approved for treating a subset of lung cancer patients [33]. Subsequent CheckMate 017 and 057 studies showed that nivolumab could improve overall survival rates compared to second‐line docetaxel in both advanced squamous and nonsquamous lung cancer patients, respectively [25]. The 2‐year OS rates were 23% with nivolumab versus 8% with docetaxel in patients with SQCC and 29% with nivolumab versus 16% with docetaxel in patients with nonsquamous NSCLC. In addition, nivolumab had a longer mean duration of treatment, a lower rate of the most common treatment‐related adverse events (any grade, 68% vs. 88%; Grade 3 or 4, 10% vs. 55%), and a lower rate of adverse events leading to discontinuation (6% vs. 13%) [25]. Subsequently, immunotherapeutic drugs that target immune checkpoint pathways have made significant strides in clinical trials and have quickly become the standard of care for advanced‐stage NSCLC.

More male NSCLC patients have a SQCC histology. The 1‐YS of SQCC and male patients lacked the first turning point due to the low mutation rate of target genes [34] and the poor effect of targeted therapy [35]. In 2011, the American Society of Clinical Oncology (ASCO) recommended routine testing for EGFR mutations in nonsquamous NSCLC patients, especially AD patients [27]. Thus, the turning point around 2014 for SQCC and male patients these patients should only be attributed to immunotherapy [25, 36]. Moreover, for female NSCLC patients, especially for those with Stage 4 AD disease, an even larger improvement in 1‐YS (1.38% annual improvement) was found in 2014 compared with 2012 (1.12% annually). This accelerated improvement can largely be attributed to the introduction of immunotherapy on top of TKI targeted therapy.

The reasons for the improved survival and reduced mortality of lung cancer are multifactorial. First, the long‐term results of smoking cessation explain the decreasing incidence. Lung cancer screening programs and improved diagnostic approaches are able to detect diseases more early on. Early detection of lung cancer may also have led to improved overall survival. Despite the recommendation by the National Lung Screening Trial (NLST) that high‐risk groups should be screened by low‐dose spiral CT, uptake has been low [37]. The influence of screening should be modest because our study covered the period between 2004 and 2019. We observed a decrease in the proportion of lung cancer with unknown stages during the period, indicating improved staging techniques. Interestingly, the proportions of Stage 4 lung cancer increased (Figure S1), possibly due to early detection of metastasis by improving and wider application of imaging techniques that could lead to stage migration. It is also possible that there was a real increase in the incidence of Stage 4 lung cancer because patients live longer than before and hence later develop metastasis. It should be noted that although an increase in the proportion among all cases, the actual incidence of Stage 4 lung cancer did not increase significantly. Furthermore, the total lung cancer incidence decreased from 44.5/100 000 person‐years in 2004 to 31.4/100 000 person‐years in 2019.

In the future, further advance and refinement of drug therapy for NSCLC may also continuously improve the survival of NSCLC patients. For example, smartly use different concomitant drugs such as corticosteroid, proton pump inhibitors, and antibiotics may further promote the efficacy of immune checkpoint inhibitors [38]. In addition, future exploration of more biomarkers such as pan‐cytokeratin and more target such as EML4‐ALK fusion protein will further improve precision and efficacy in the treatment in of NSCLC patients [39].

Our study benefited from the use of large population‐based cancer registry datasets. In particular, we included lung cancer cases diagnosed from 2004 to 2019, a period over which histological classification of lung cancer remained largely consistent [40]. Treatment data were incomplete in SEER database; thus, we could not establish the causal relationship between improved therapy and survival. In addition, although minimal in SEER, the inaccuracies and misclassifications in registry data have also impacted the results [41]. However, this large population‐based registry data analysis provides optimal real‐world evidence (RWE) and helps confirm what observed in clinical trials happen in real life. This RWE have much better external validity than the results from traditional clinical trials that were conducted under strictly controlled environments. Therefore, our results harbor unique unitality for understanding the drug epidemiology and proving the effectiveness of immunotherapy in Stage 4 NSCLC at population level.

Overall survival represents the best indicator for clinical management and is more informative than incidence or mortality for clinicians and patients. Survival changes at the population level might inform the public that genuine breakthroughs have occurred in management. This study, for the first time, finds that immunotherapy significantly improves the survival of Stage 4 NSCLC at the US population level. Moreover, ICIs and TKIs have recently been shown to be effective for early‐ and locally advanced‐stage disease [42]. With the continuous advancement, targeted therapy and immunotherapy will definitely improve the survival for NSCLC of all stages in the near future.

## 5 Conclusion

Through the change in survival trends over the past 15 years, our study revealed two significant turning points in the survival of lung cancer in the United States: one around 2010 and another around 2014, which were associated with the introduction of molecular targeted therapy and immunotherapy, respectively. The accelerated increasing survival of NSCLC patients after 2014 at the population level indicates that immunotherapy has truly revolutionized the management of NSCLC. Our findings provide population‐level evidence supporting the use of immunotherapy, which might have profound implications for health care policy in lung cancer.

## Author Contributions

Conceptualization: Z.F. Methodology: Z.F., Y.W., R.Z., and J.H. Validation: Y.W., R.Z., and J.H. Formal analysis, Y.W., R.Z., J.H., R.C., and S.W. Investigation: Y.W., R.Z., R.Y., and J.H. Resources: Y.W., R.Z. and Z.F. Data curation, Y.W., R.Z., R.Y., and J.H. Writing – original draft preparation: All authors. Writing – review and editing: All authors. Visualization: Y.W. Supervision: Z.F. Project administration: Z.F. All authors have read and agreed to the published version of the manuscript.

## Ethics Statement

Patient consents were not required because the study is a retrospective database research in nature, and there was no direct patient contact. Institutional Review Board approval was not required according to our institution’s policy.

## Conflicts of Interest

The authors declare no conflicts of interest.

## Supporting information


**Figure S1.** Distribution of Stages and Stage‐Specific Survival Trends for NSCLC and SCLC.
**Figure S2.** Distribution of Stages and Different Treatment Modalities in NSCLC and SCLC.

## Data Availability

All data used in this study are publicly available in the Surveillance, Epidemiology, and End Results at https://seer.cancer.gov.
