# Supplementary material for: Immunotherapy Improves the Survival of Stage 4 Non–Small Cell Lung Cancer Patients at the US Population Level: The Real‐World Evidence
Source: Clin Respir J. 2024 Sep 14;18(9):e70000. doi: 10.1111/crj.70000 (PMC11399776; doi:10.1111/crj.70000)
Supplement: Supplementary file 1 — Figure S1. Distribution of Stages and Stage‐Specific Survival Trends for NSCLC and SCLC. Figure S2. Distribution of Stages and Different Treatment Modalities in NSCLC and SCLC. [file CRJ-18-e70000-s001.pdf]

Supplemental Content

Figure S1. Distribution of Stages and Stage-Specific Survival Trends for NSCLC and SCLC

Figure S2. Distribution of Stages and Different Treatment Modalities in NSCLC and SCLC

This supplemental material has been provided by the authors to give readers additional information about their work.

Figure S1. Distribution of Stages and Stage-Specific Survival Trends for NSCLC and SCLC

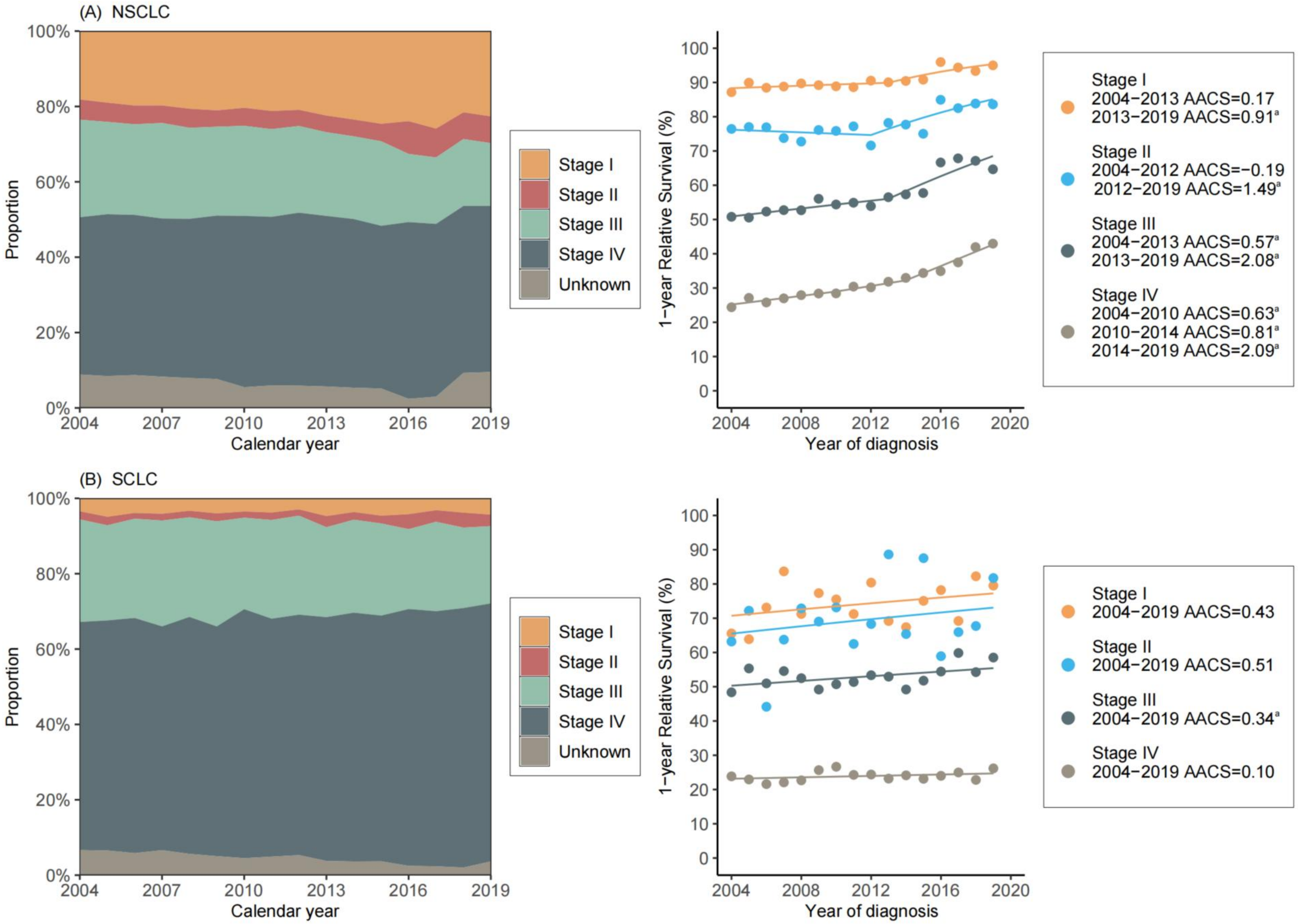

Abbreviations: NSCLC, non-small cell lung cancer; SCLC, small cell lung cancer.

The percentage associated with each line represents the average absolute change in survival (AACS) during the indicated range of years.

[a] *P* <.05.

Figure S2. Distribution of Stages and Different Treatment Modalities in NSCLC and SCLC

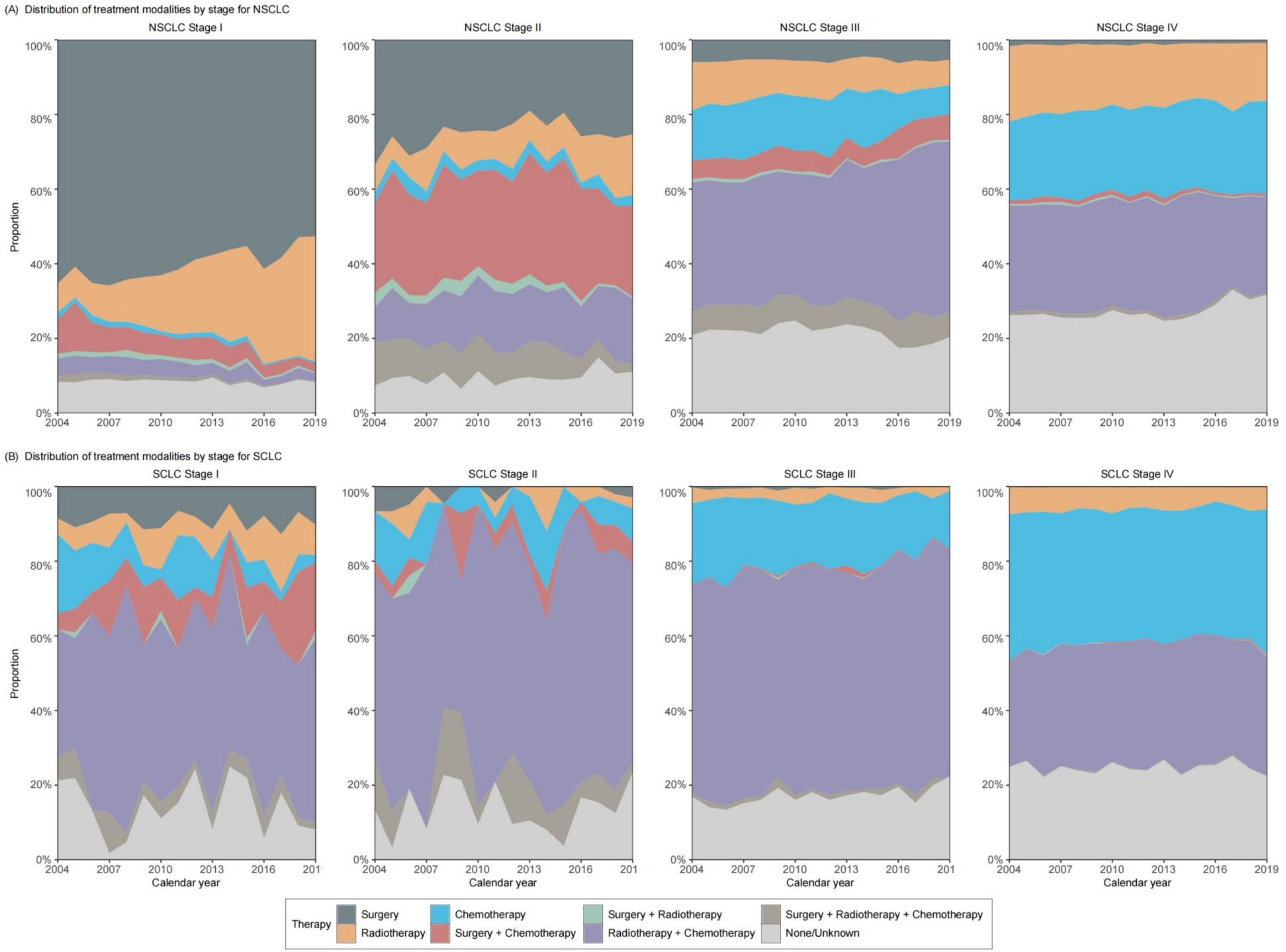

Abbreviations: NSCLC, non-small cell lung cancer; SCLC, small cell lung cancer.
